# Mortality among individuals exposed to atomic bomb radiation in utero: 1950–2012

**DOI:** 10.1007/s10654-020-00713-5

**Published:** 2021-01-25

**Authors:** Hiromi Sugiyama, Munechika Misumi, Ritsu Sakata, Alina V. Brenner, Mai Utada, Kotaro Ozasa

**Affiliations:** 1 Department of Epidemiology, Radiation Effects Research Foundation, 5-2 Hijiyama Park, Minami-ku, Hiroshima City, Hiroshima Japan; 2 Department of Statistics, Radiation Effects Research Foundation, 5-2 Hijiyama Park, Minami-ku, Hiroshima City, Hiroshima Japan

**Keywords:** Atomic bomb survivors, In utero exposure, Radiation risk, Mortality, Birth weight

## Abstract

We examined the mortality risks among 2463 individuals who were exposed in utero to atomic bomb radiation in Hiroshima or Nagasaki in August 1945 and were followed from October 1950 through 2012. Individual estimates of mother’s weighted absorbed uterine dose (DS02R1) were used. Poisson regression method was used to estimate the radiation-associated excess relative risk per Gy (ERR/Gy) and 95% confidence intervals (CI) for cause-specific mortality. Head size, birth weight, and parents’ survival status were evaluated as potential mediators of radiation effect. There were 339 deaths (216 males and 123 females) including deaths from solid cancer (n = 137), lymphohematopoietic cancer (n = 8), noncancer disease (n = 134), external cause (n = 56), and unknown cause (n = 4). Among males, the unadjusted ERR/Gy (95% CI) was increased for noncancer disease mortality (1.22, 0.10–3.14), but not for solid cancer mortality (− 0.18, < − 0.77–0.95); the unadjusted ERR/Gy for external cause mortality was not statistically significant (0.28, < − 0.60–2.36). Among females, the unadjusted ERRs/Gy were increased for solid cancer (2.24, 0.44–5.58), noncancer (2.86, 0.56–7.64), and external cause mortality (2.57, 0.20–9.19). The ERRs/Gy adjusted for potential mediators did not change appreciably for solid cancer mortality, but decreased notably for noncancer mortality (0.39, < − 0.43–1.91 for males; 1.48, − 0.046–4.55 for females) and external cause mortality (0.10, < − 0.57–1.96 for males; 1.38, < − 0.46–5.95 for females). In conclusion, antenatal radiation exposure is a consistent risk factor for increased solid cancer mortality among females, but not among males. The effect of exposure to atomic bomb radiation on noncancer disease and external cause mortality among individuals exposed in utero was mediated through small head size, low birth weight, and parental loss.

**Supplementary Information:**

The online version of this article (10.1007/s10654-020-00713-5) contains supplementary material, which is available to authorized users.

## Introduction

Radiation has been recognized to increase the risks of mortality (followed-up through age of 46 years) [1] and incidence of adult-onset solid cancer (followed-up through age of 55 years) in individuals who were acutely exposed to atomic bomb radiation in utero in Hiroshima and Nagasaki, Japan [2–4]. However, the studies of those chronically exposed in utero in the Southern Urals of Russia, residents of communities near the contaminated Techa River and offspring of Mayak nuclear workers, did not show increased risks of incidence and mortality for solid cancer [5] and no increased risks of incidence or mortality for hematological malignancy in adulthood [6].

Little evidence is available on the radiation-associated risk of noncancer diseases among individuals exposed in utero. These risks have remained controversial even among in utero cohorts of atomic bomb survivors. The number of noncancer disease deaths during 1950–1984 among those aged 4–39 years and exposed to ≥ 0.6 Gy (10 cases) was significantly higher than expected [2]. However, there were no significant associations of radiation dose with hypertension, hypercholesterolemia or cardiovascular disease (CVD) among a clinical subcohort of in utero exposed cohort members aged 33–58 years [7]. A comprehensive interpretation of radiation-associated effects of noncancer diseases among in utero-exposed people remains elusive.

**Fig. 1:**
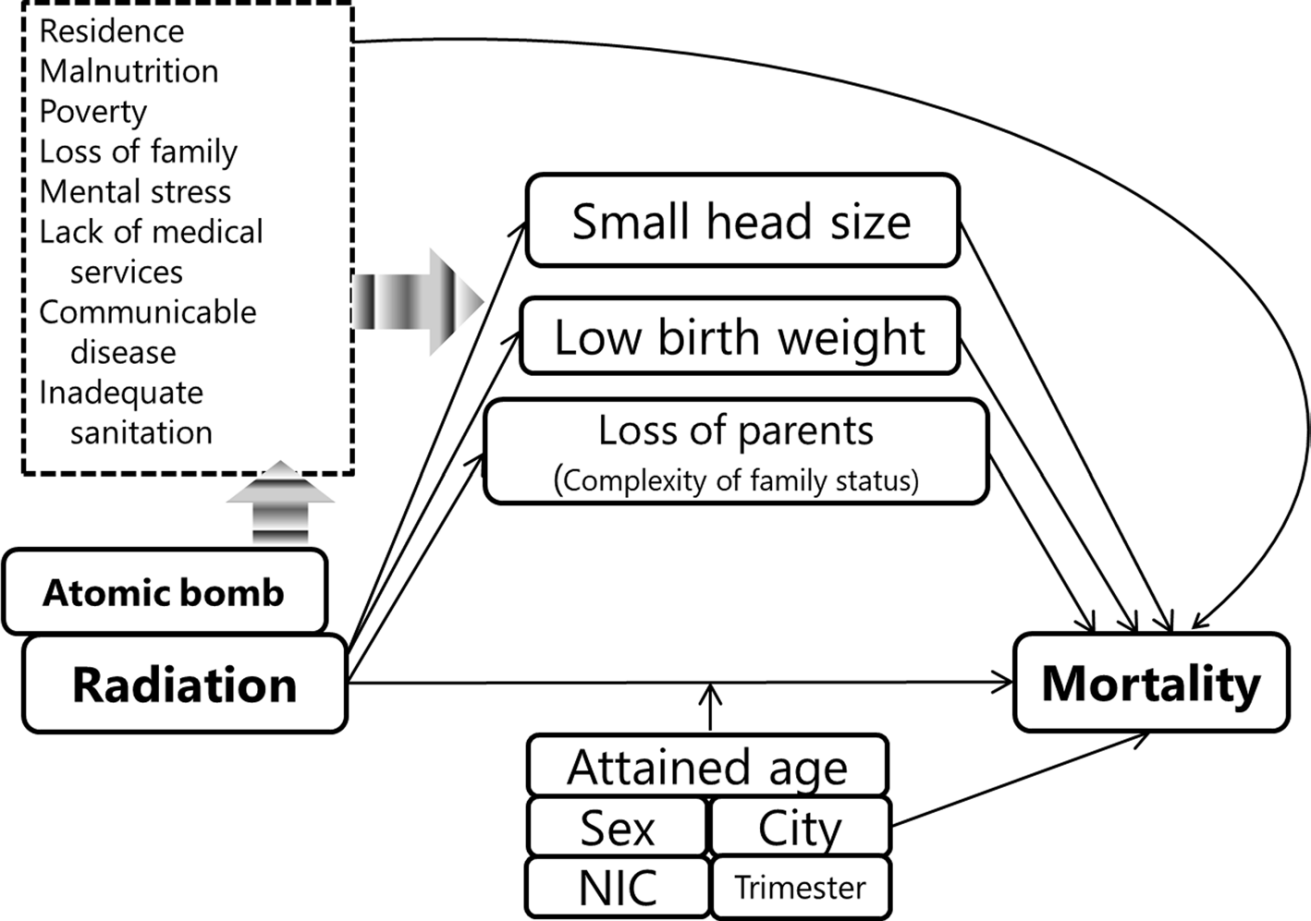
Potential relationship among atomic bomb radiation, observed and unobserved factors and mortality of individuals exposed in utero is illustrated. Factors framed with solid line indicates observed variables and those framed with dashed line indicates unobserved variables. When atomic bomb radiation is examined to affect mortality among individuals exposed in utero, attained age, sex, city, NIC (whether subjects were in either Hiroshima or Nagasaki City at the time of bombing), and trimester at the time of bombing are examined as factors to describe background mortality and to modify radiation risk of mortality. Small head size, low birth weight and loss of parents as a proxy of complex of family status were assumed to be potential mediators from exposure to atomic bomb radiation to risk of deaths. Several unobserved factors framed with dashed line such as residence, malnutrition, poverty, loss of family (e.g., grandparents, siblings and relatives), mother’s mental stress during pregnancy, lack of medical services, communicable diseases, and inadequate sanitation might affect these three observed factors and mortality

To evaluate the lifetime mortality risk associated with birth following exposure to atomic bomb radiation, Fig. 1 illustrates the potential relationship among atomic bomb radiation, observed and unobserved factors and mortality. The frequency of small head size showed a radiation dose response in individuals exposed to atomic bomb radiation in utero [8]. The prevalence of low birth weight was high among those who were exposed in proximal areas (0–1499 m from the hypocenters) [9]. Many individuals were exposed in utero in the proximal areas and lost one or both of their parents [9]. The observed factors (i.e., small head, low birth weight, loss of parents) could be explained by unmeasured factors, such as size and area of their residence, malnutrition, poverty, loss of family members (i.e., siblings, grandparents, relatives), mental stress during pregnancy, lack of access to medical services, communicable diseases, and inadequate sanitary facilities because the atomic bombings caused not only radiation exposure but also exposure to heat and blast, and the substantial destruction of homes and social infrastructure. However, it is still unclear whether and how these observed factors affected mortality for individuals exposed in utero. These factors might be explained as social or biological mediators, which might be caused by atomic bombs and might affect mortality for individuals exposed in utero. Thus, we have to carefully assess causal pathways to the outcome, i.e., mortality, to determine if there are direct effects of radiation exposure or other influences of atomic bombings.

We aimed to examine the radiation effects on mortality by a subgroup of causes of death, considering potential mediators such as birth weight, head size, and parental survival status among individuals aged 4–67 years during 1950–2012 following exposure to atomic bombs in utero.

## Methods

### Study cohort and subjects

The in utero cohort comprised 3638 members born to mothers exposed to atomic bomb radiation and who were born after the bombing between August 6, 1945, in Hiroshima or August 9, 1945, in Nagasaki and May 31, 1946. They originated from two precedent study programs: clinical (n = 1606) [8, 10] and mortality (n = 2802) [9] studies. Some members belonged to both programs (n = 770). Although details are explained in a RERF Technical Report by Schull and Otake [11], the summary of the two programs is described briefly below.

1. *Clinical study program* Members were alive as of October 1, 1950, and lived in contact areas in Hiroshima and Nagasaki according to the Atomic Bomb Casualty Commission (ABCC, the former organization of RERF). They included all individuals who were exposed to the atomic bomb within 2000 m of the hypocenter, and those who were exposed between 3000 and 4999 m, or those who were located beyond 10,000 m at the time of the bombing. Members of the latter two groups were matched by sex and month of birth to the former group [8, 10]. The members of this cohort were finally fixed in July 1959 [10]. The members were followed up clinically until they reached 19 years of age.
2. *Mortality study program* Members were children born to mothers who were exposed within 1500 m of the hypocenter or beyond 1500 m. The latter group was matched to the former group by city, sex, and month of birth. Members were selected from the master file list, which is a central file recording of all individuals known to the ABCC, and birth reports from Hiroshima and Nagasaki Cities. The subjects were followed from the date of birth. Another 461 members were added after the 1960 National Census. They have been followed since 1960. The detailed sampling methods are described elsewhere [9]. A self-administered questionnaire on socioeconomic status was conducted by mail in 1964 [9].

Of all cohort members (n = 3638), those with no information in the family registry system of Japan (*koseki*) (n = 18), those with foreign nationality (n = 55), those registered in duplicate (n = 9), those born to mothers whose radiation dose was not estimated (n = 879), and those who died before the start of follow-up (n = 214) were excluded. Finally, the number of eligible subjects for the analysis was 2463.

Vital status was identified through *koseki*. Follow-up started on October 1, 1950 for those selected based on birth reports, the date of entry in the ABCC master file for those selected based on the master file (because of lack of birth reports), July 15, 1959 for those derived from the clinical program only, or October 1, 1960 for those selected based on the 1960 National Census, and ended on the date of death, the date of loss to follow-up, or December 31, 2012, whichever occurred first. The underlying cause of death was determined via the death certificate and was coded using the International Classification of Diseases (ICD) versions 7–10; the codes were converted to the ICD version 9.

### Exposure and potential mediators

Individual radiation dose was the sum of absorbed doses from neutrons and gamma rays, weighted by a biological effectiveness factor of 10 for neutrons, and was estimated based on the Dosimetry System 2002 Revision 1 (DS02R1) [12]. The mother’s weighted absorbed uterine dose was used as a proxy due to the lack of the fetal dose estimate in the DS02R1.

Data of trimester of pregnancy at the time of bombing was obtained from the master information of the clinical program and the mail-based socioeconomic survey of the mortality program. Trimesters (1st, 2nd, or 3rd) in both datasets were calculated using the same methods below [11].

$$ {\text{Days of pregnancy at the time ofbombing}} = 2 80\;{-}\;\left( {{\text{date of birth}}\;{-}\;{\text{August 6 or 9}}, 1 9 4 5} \right), $$

where the mean duration of pregnancy was taken to be 280 days. The date of birth was based on the date obtained in interviews with the clinical cohort subjects or their mothers by Otake and Schull [8] or obtained by the socioeconomic survey among mortality cohort members [9]. Neither study used the date of birth on the birth reports found in *koseki*. Trimesters of pregnancy at the time of the bombing were defined as follows for Hiroshima and in parenthesis for Nagasaki.

- 1st trimester: 7 (10) February 1946 to 31 May 1946
- 2nd trimester: 7 (10) November 1945 to 6 (9) February 1946
- 3rd trimester: 6 (9) August 1945 to 6 (9) November 1945

Information on birth weight was obtained by interviews in the clinical program [8] and the socioeconomic survey in the mortality program [9]. Birth weight was classified into low (< 2500 g), normal (≥ 2500 g), unknown (those who were surveyed in either program but whose information was insufficient), and no information (those who were not surveyed in either program).

Of the 1566 clinical program subjects, 1473 had their head circumference measured at least once between the ages of 9 and 19 years in the original clinical program [8]; 62 patients with small head (i.e., smaller than 2 standard deviations from sex- and age-specific mean) were identified from the internal RERF documents and treated as individuals with small head size in this study. We could not identify additional three subjects with small head size who died before the age of 9 years as reported by Wood et al. [13]. We could not identify whether the other subjects had a normal head size or their head circumference was not measured. The clinical cohort included all individuals who were exposed within 2000 m from the hypocenters and resided in Hiroshima or Nagasaki in 1950; thus, we treated all individuals other than 62 patients with small head, including three individuals with small head size who died before the measurement and those derived only from the mortality program, as individuals with normal head size.

Information on biological parental survival status was obtained by the socioeconomic survey in 1964 that was conducted for mortality program members [9] and used in the analysis as a proxy for the complexity of family status. The category “loss” included death, divorce, or disappearance due to any reason, including war. Subjects derived only from the clinical program were treated as having “no information.”

### Statistical analysis

The associations between radiation dose and potential mediators were examined, by a logistic regression model, with head size as a binary response variable, and multinomial logistic regression models, with birth weight and parental survival status as response variables. The odds ratio (OR) of small head circumference and relative odds ratio (RORs) of low birth weight, loss of father, and loss of mother were estimated with adjustment for city, sex, exposure to atomic bombings (NIC: not in city vs. in city), cohort source (birth reports, master file, or 1960 National Census), and trimester at the time of bombing (1st, 2nd, or 3rd).

The area within 2000 m of the hypocenter was exposed to high-dose radiation (mean dose: 318 mGy, range: 19 mGy to 2.52 Gy), but, in addition, all houses and buildings were burnt by the bombings. The areas between 2000 and 3000 m from the hypocenters were where radiation exposure was not thought to be remarkable (mean dose: 8.6 mGy, range: 0.5–54 mGy), but the infrastructure was destroyed. To examine the geographical influence due to the destruction of social resources on potential mediators, we examined the frequency of each potential mediator by distance from the hypocenter.

The number of deaths was observed for subsets of causes of death. A Poisson regression model based on “ungrouped” data, the survival data of individuals stratified by attained age (4–9 years, and 2-year intervals for those older than 10 years) and follow-up period divided into 5-year intervals was used to estimate the background mortality rates and the radiation-associated excess relative risk per 1 Gy (ERR/Gy) for solid cancer (ICD 9th: 140–199), noncancer disease (ICD 9th: 001–139, 240–779), and external cause (ICD 9th: 800–999) mortality. Tests of significance and estimations of 95% confidence intervals (CIs) were based on likelihood ratio statistics and direct evaluation of the profile likelihood. Radiation effects and background mortality rates were described based on linear ERR models.

The basic model was as follows:

$$\lambda = \lambda_{0} \left( {c,s,c*nic,a,cs,t} \right)*\left[ { 1+ {\text{ERR}}\left( d \right)} \right], $$

where background mortality rate (λ_0_), namely, the rate for those who were not exposed to a radiation dose, was modeled as a function of city (*c*), sex (*s*), location at the time of bombing (*nic*) (NIC vs. in city), log^2^(attained age/20 for age < 20 years), log(attained age/60), log^2^(attained age/60) (*a*), cohort source (*cs*), and trimester (*t*) (1st, 2nd, or 3rd).

The full model was as follows:

$$ \lambda = \lambda_{0} \left( {c,s,c*nic,a,cs,t,w,s*h,fs,ms} \right)*\left[ { 1+ {\text{ERR}}\left( d \right)} \right], $$

where the ERR was additionally adjusted for birth weight (*w*), head size (*h*), and father (*fs*) or mother’s survival status (*ms*).

In Japan, infant mortality rates were extremely high for 15 years after World War II [14], and major underlying causes of death in children differed between that period and thereafter. Thus, background mortality rates and ERR/Gy estimates of radiation-associated death were evaluated based on the cases identified during the early follow-up period and those in adulthood (i.e., older than the attained age of 20 years).

The person-years, background mortality rates and ERRs/Gy were estimated using EPICURE 2.00.02 software (DATAB and AMFIT modules, Risk Sciences International, Inc. Ottawa, Canada. https://www.risksciences.com/project/epicure/) and R version 3.6.1 (R Foundation, Vienna, Austria. https://www.r-project.org/foundation/).

## Results

We observed 2463 subjects, with a total of 138,003 person-years of follow-up through 2012 (Table 1). A total of 2121 (86.1%) subjects were alive at the end of the follow-up period. The mean radiation dose was 123 mGy (range 0 Gy to 2519 mGy) among those excluding the NIC group. The frequency of individuals with low birth weight (11.7% = 84/715), those with small head size (6.1% = 44/715), and those who lost fathers (31.6% = 226/715) were high for subjects exposed within 2000 m of the hypocenters compared to subjects exposed at other distances (Table 2). Atomic bomb radiation had a highly significant association with low birth weight (Table 3). Small head size and loss of father were also significantly associated with radiation dose, but loss of mother was not significantly associated (Table 3).

**Table 1:** Number of subjects and person-years

	Mother’s weighted absorbed uterine dose (Gy)						Person-years
	Not in city	< 0.005	< 0.1	< 1	≥ 1	Total	
Mean dose (Gy)		0.001	0.037	0.327	1.635	0.123	
	N (%)	N (%)	N (%)	N (%)	N (%)	N (%)	
Sex							
Male	284 (48.5)	479 (49.4)	214 (49.5)	213 (49.4)	24 (53.3)	1214 (49.3)	67,437
Female	301 (51.5)	491 (50.6)	218 (50.5)	218 (50.6)	21 (46.7)	1249 (50.7)	70,567
City							
Hiroshima	508 (86.8)	759 (78.2)	383 (88.7)	364 (84.5)	31 (68.9)	2045 (83.0)	115,058
Nagasaki	77 (13.2)	211 (21.8)	49 (11.3)	67 (15.5)	14 (31.1)	418 (17.0)	22,945
Cohort sources							
1960 Census	22 (3.8)	171 (17.6)	42 (9.7)	54 (12.5)	6 (13.3)	295 (12.0)	14,905
Birth report	212 (36.2)	393 (40.5)	280 (64.8)	298 (69.1)	35 (77.8)	1218 (49.5)	72,207
Master file	351 (60.0)	406 (41.9)	110 (25.5)	79 (18.3)	4 (8.9)	950 (38.6)	50,891
Trimester							
1st trimester	164 (28.0)	340 (35.1)	139 (32.2)	126 (29.2)	13 (28.9)	782 (31.7)	43,381
2nd trimester	222 (37.9)	351 (36.2)	162 (37.5)	175 (40.6)	20 (44.4)	930 (37.8)	52,333
3rd trimester	199 (34.0)	280 (28.9)	131 (30.3)	130 (30.2)	12 (26.7)	751 (30.5)	42,289
Birth weight							
Normal (≥ 2500 g)	516 (88.2)	716 (73.8)	357 (82.6)	343 (79.6)	23 (51.1)	1955 (79.4)	110,549
Low (< 2500 g)	45 (7.7)	59 (6.1)	38 (8.8)	47 (10.9)	17 (37.8)	206 (8.4)	11,380
Unknown^a^	19 (3.2)	33 (3.4)	17 (3.9)	31 (7.2)	5 (11.1)	105 (4.3)	5747
No information^b^	5 (0.9)	162 (16.7)	20 (4.6)	10 (2.3)	0 (0.0)	197 (8.0)	10,327
Head size^c^							
Male, normal head size	282 (99.3)	475 (99.2)	213 (99.5)	196 (92.0)	17 (70.8)	1183 (97.4)	65,832
Male, small head size^d^	2 (0.7)	4 (0.8)	1 (0.5)	17 (8.0)	7 (29.2)	31 (2.6)	1605
Female, normal head size	295 (98.0)	486 (99.0)	212 (97.2)	208 (95.4)	18 (85.7)	1219 (97.6)	68,959
Female, small head size^e^	6 (2.0)	5 (1.0)	6 (2.8)	10 (4.6)	3 (14.3)	30 (1.2)	1607
Father’s survival status							
Alive	264 (45.1)	487 (50.2)	280 (64.8)	255 (59.2)	21 (46.7)	1307 (53.1)	75,521
Lost	50 (8.5)	146 (15.1)	101 (23.4)	146 (33.9)	24 (53.3)	467 (19.0)	26,632
Mother’s survival status							
Alive	288 (49.2)	578 (59.6)	347 (80.3)	360 (83.5)	40 (88.9)	1613 (65.5)	93,249
Lost	26 (4.4)	55 (5.7)	34 (7.9)	41 (9.5)	5 (11.1)	161 (6.5)	8904
Parent’s survival status							
No information	271 (46.3)	337 (34.7)	51 (11.8)	30 (7.0)	0 (0.0)	689 (28.0)	35,850
Total	585 (100)	970 (100)	432 (100)	431 (100)	45 (100)	2463 (100)	138,003

^a^Unknown: Individuals who were surveyed but of whom information was insufficient in either the clinical program and the socioeconomic survey

^b^No information: Individuals who were not surveyed in any programs

^c^Sixty-two subjects with small heads were identified from the clinical program internal documents. Individuals other than 62 subjects with small heads, three individuals with small head size who died before the measurement, and those derived only from the mortality program were treated as individuals with normal head size

^d^Among 31 male subjects with small head size, 16 individuals were exposed in the 1st trimester, 9 individuals in the 2nd trimester, and 6 individuals in the 3rd trimester

^e^Among 30 female subjects with small head size, 18 individuals were exposed in the 1st trimester, 10 individuals in the 2nd trimester, and 2 individuals in the 3rd trimester

**Table 2:** Number of subjects and proportion (%) by birthweight, head size, and parent’s survival status by distance from hypocenters

	Distance from the hypocenters				
	< 2000 m	< 3000 m	≥ 3000 m	NIC	Total
Birth weight					
Normal (≥ 2500 g)	559 (78.2)	323 (86.4)	557 (70.6)	516 (88.2)	1955 (79.4)
Low (< 2500 g)	84 (11.7)	28 (7.5)	49 (6.2)	45 (7.7)	206 (8.4)
Unknown^a^	47 (6.6)	18 (4.8)	21 (2.7)	19 (3.2)	105 (4.3)
No information^b^	25 (3.5)	5 (1.3)	162 (20.5)	5 (0.9)	197 (8.0)
Head size					
Male, nomal head size	330 (93.0)	192 (100.0)	379 (99.0)	282 (99.3)	1183 (97.4)
Male, small head size	25 (7.0)	0 (0.0)	4 (1.0)	2 (0.7)	31 (2.6)
Female, nomal head size	341 (94.7)	182 (100.0)	401 (98.8)	295 (98.0)	1219 (97.6)
Female, small head size	19 (5.3)	0 (0.0)	5 (1.2)	6 (2.0)	30 (2.4)
Father’s survival status					
Alive	416 (58.2)	268 (71.7)	359 (45.5)	264 (45.1)	1307 (53.1)
Lost	226 (31.6)	89 (23.8)	102 (12.9)	50 (8.5)	467 (19.0)
Mother’s survival status					
Alive	576 (80.6)	323 (86.4)	426 (54.0)	288 (49.2)	1613 (65.5)
Lost	66 (9.2)	34 (9.1)	35 (4.4)	26 (4.4)	161 (6.5)
Parent’s survival status					
No information	73 (10.2)	17 (4.5)	328 (41.6)	271 (46.3)	689 (28.0)
Total	715	374	789	585	2463

^a^Unknown: Individuals who were surveyed but of whom information was insufficient in either the clinical program and the socioeconomic survey

^b^No information: Individuals who were not surveyed in any programs

**Table 3:** Relationship between radiation dose and potential mediators

	ROR^c^ at 1 Gy	95% CI^e^
Birth weight		
≥ 2500 g	Ref.^f^	
< 2500 g	3.47	(2.37–5.08)
Unknown^a^	2.84	(1.74–4.65)
No information^b^	0.00	(0.00–0.01)

	OR^d^ at 1 Gy	95% CI^e^
Head size		
Normal	Ref.^f^	
Small	5.16	(3.18–8.38)

	ROR^c^ at 1 Gy	95% CI^e^
Father’s status		
Alive	Ref.^f^	
Lost	2.27	(1.63–3.17)
No information	0.00	(0.00–0.13)
Mother’s status		
Alive	Ref.^f^	
Lost	1.31	(0.83–2.06)
No information	0.00	(0.00–0.02)

Multinomial logistic regression was applied to estimate the RORs of birth weight and parental survival status, while logistic regression was used to estimate the OR of small head size

Radiation dose was adjusted for sex, city, cohort source, location at time of bombing, and trimester

^a^Unknown: Individuals who were surveyed but of whom information was insufficient in either the clinical program and the socioeconomic survey

^b^No information: Individuals who were not surveyed in any programs

^c^ROR: Relative odds ratio

^d^OR: Odds ratio

^e^CI: Confidence interval

^f^Ref.: reference

### Observed deaths

A total of 339 deaths (13.8% of the cohort), including solid cancer deaths (n = 137, 40.4% of the deaths), lymphohematopoietic cancer deaths (n = 8, 2.4%), noncancer disease deaths (n = 134, 39.5%), external cause deaths (n = 56, 16.5%), and unknown cause deaths (n = 4, 1.6%) were observed (Table 4). Because the number of lymphohematopoietic cancer deaths was limited, we did not estimate background mortality rate and the radiation-associated ERR.

**Table 4:** Number of deaths by cause of death and by dose category among individuals exposed to atomic bomb radiation in utero

Cause of death	Males						Females					
	Mother’s weighted absorbed uterine dose (Gy)						Mother’s weighted absorbed uterine dose (Gy)					
	NIC	< 0.005	< 0.1	< 1	≥ 1	Total	NIC	< 0.005	< 0.1	< 1	≥ 1	Total
All causes	49	90	34	36	7	216	27	41	17	28	10	123
Solid cancer	22	34	13	10	1	80	13	23	5	12	4	57
Lip, oral cavity	2	0	1	0	0	3	0	0	0	0	0	0
Esophagus	0	2	1	2	0	5	2	1	0	0	0	3
Stomach	4	11	1	0	1	17	1	2	2	5	0	10
Colon	0	3	0	2	0	5	3	2	0	0	0	5
Rectum	2	3	0	2	0	7	0	0	0	0	0	0
Liver	6	5	3	1	0	15	2	1	0	0	1	4
Gallbladder	0	1	1	0	0	2	2	2	0	0	0	4
Pancreas	3	0	0	1	0	4	1	1	0	1	0	3
Gastrointestinal tract, NOS	1	1	0	0	0	2	0	0	0	0	0	0
Larynx	0	1	0	0	0	1	0	0	0	0	0	0
Lung	4	5	5	1	0	15	1	3	1	1	0	6
Heart	0	0	0	0	0	0	0	0	1	0	0	1
Mesothelioma	0	0	0	1	0	1	0	0	0	0	0	0
Breast	0	0	0	0	0	0	0	4	0	3	1	8
Vulva							0	1	0	0	0	1
Vagina							0	0	0	1	0	1
Cervix uteri							0	2	0	0	0	2
Corpus uteri							0	0	1	0	0	1
Uterus NOS							0	2	0	0	0	2
Ovary							0	0	0	1	1	2
Prostate	0	0	1	0	0	1						
Kidney	0	1	0	0	0	1	0	0	0	0	0	0
Renal pelvis	0	1	0	0	0	1	0	0	0	0	0	0
Brain	0	0	0	0	0	0	1	0	0	0	0	1
Thyroid	0	0	0	0	0	0	0	1	0	0	0	1
Second malignancy	0	0	0	0	0	0	0	1	0	0	1	2
Lymphohematopoietic cancer	0	0	2	0	0	2	0	2	2	2	0	6
Lympohoma and Myeloma	0	0	0	0	0	0	0	1	0	2	0	3
Leukemia	0	0	2	0	0	2	0	1	2	0	0	3
Noncancer disease	24	34	13	18	5	94	9	11	6	10	4	40
Infectious disease	0	4	2	3	0	9	2	1	0	0	3	6
Blood disease	1	0	0	0	0	1	0	0	0	0	0	0
Diabetes	0	0	1	3	0	4	0	0	1	0	0	1
Mental disorders	0	0	1	0	0	1	0	0	0	0	0	0
Nerve system	1	1	0	1	1	4	0	3	0	0	0	3
Circulatory disease	10	13	6	9	1	39	5	3	4	4	1	17
Respiratory disease	3	5	1	0	1	10	1	0	0	1	0	2
Digestive diseases	7	3	1	2	1	14	0	0	0	1	0	1
Genitourinary disease	0	3	0	0	1	4	1	2	0	2	0	5
Other disease	2	5	1	0	0	8	0	2	1	2	0	5
External causes	3	20	6	6	1	36	5	5	4	4	2	20
Suicide	1	12	4	1	1	19	3	2	2	2	1	10
Accident	1	6	2	4	0	13	2	3	1	2	0	8
Unknown	0	2	0	2	0	4	0	0	0	0	0	0
Person-years	15,722	25,943	12,320	12,181	1271	67,437	16,890	27,419	12,734	12,476	1047	70,567

NIC: Not in city at the time of atomic bombings, NOS: Not otherwise specified

### Background mortality rate

**Fig. 2:**
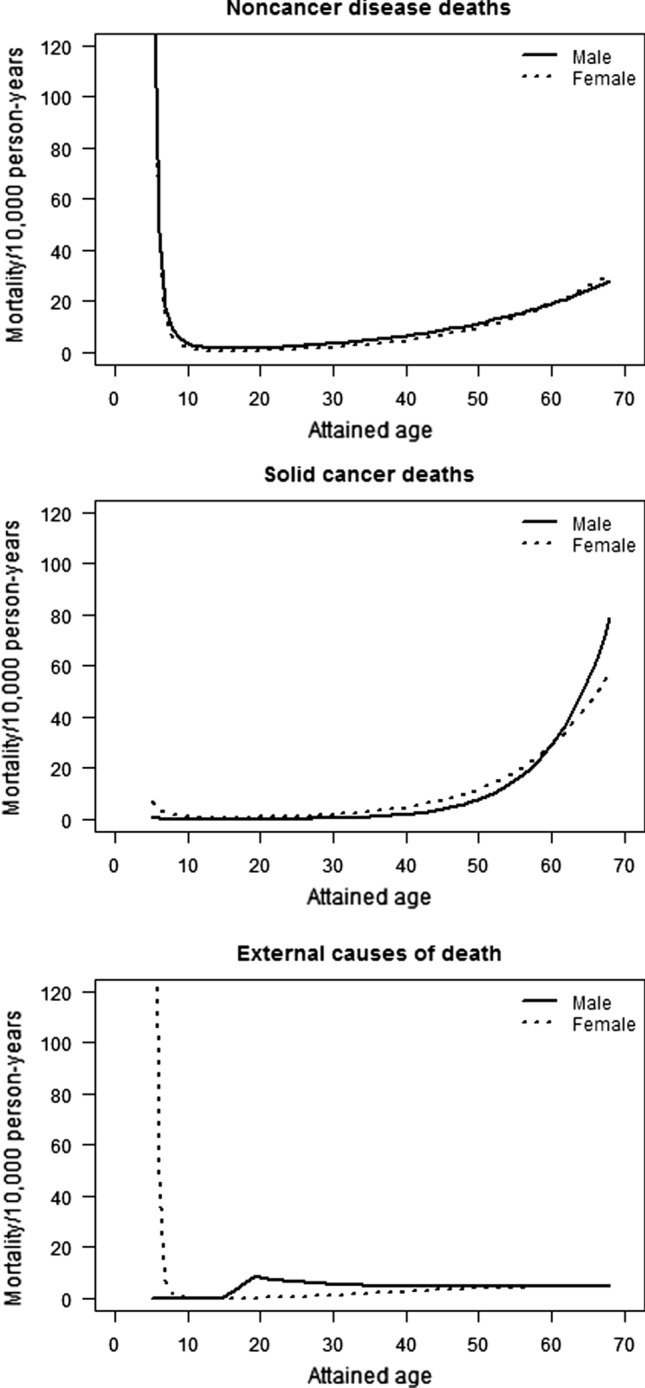
Sex-specific background rates by cause of death among those exposed to atomic bomb radiation in utero

Solid cancer mortality rates in females increased after the attained age of 20 years, and the rates in males increased after the age of 40 years (Fig. 2). The high background mortality rates for noncancer disease for both sexes (e.g., infectious disease) and external cause deaths in females (e.g., accidental drowning) were observed during childhood (Fig. 2). The rates of noncancer disease mortality in both males and females increased with an attained age of 30 years or older. In contrast, the rates of external cause of death remained approximately constant in those aged 20 years or older (Fig. 2). The background mortality rates for noncancer disease and external causes by trimester did not vary (data not shown), whereas the background mortality rates for solid cancer for exposure in the 2nd trimester was decreased (relative risk (RR) with respect to 3rd trimester = 0.58: 95% CI: 0.38–0.89).

### Radiation-associated ERR and the change in ERR adjusted for potential mediators (adjusted ERR)

#### Solid cancer

None of the potential mediators was associated with background mortality rates for solid cancer either among males or females. Neither unadjusted nor adjusted male ERR/Gy for solid cancer deaths was increased (Table 5). The unadjusted female ERR/Gy was increased, at 2.24 (95% CI: 0.44–5.58); the adjusted ERR/Gy increased by 12% to 2.51 (95% CI: 0.53–6.28). The adjusted ERR/Gy for adulthood (≥ 20 years of age) solid cancer mortality was unaltered for males and remained significantly raised for females (2.10, 95% CI: 0.26–5.61). To investigate sensitivity of the unadjusted ERR/Gy for adulthood solid cancer mortality especially for the assessment of different radiation risks between sexes, selected cancer sites (i.e., stomach, lung, liver, breast, and sex-specific cancers) were evaluated and the unadjusted ERRs/Gy for cancers excluding selected cancer sites were estimated. The estimates of ERRs/Gy for deaths due to all solid cancer, cancers of some sites (stomach, liver, and breast), and cancers of sex-specific sites as a whole (i.e., breast, vulva, vagina, uterus, ovary, and prostate) showed increasing radiation risks, but were not statistically significant (Supplementary Table 1). The ERR/Gy did not differ by trimester at the time of bombing or attained age.

**Table 5:** Radiation ERRs/Gy and relative risks of potential mediators on mortality among individuals exposed to atomic bomb radiation in utero

	Solid cancer deaths		Noncancer disease deaths		External causes of death	
	Male	Female	Male	Female	Male	Female
*All attained age*						
ERR/Gy (95% CI) Not adjusted for potential mediators^a^	− 0.18 (< − 0.77–0.95)	2.24 (0.44–5.58)	1.22 (0.10–3.14)	2.86 (0.56–7.64)	0.28 (< − 0.60–2.36)	2.57 (0.20–9.19)
ERR/Gy (95% CI) Adjusted for potential mediators^b^	− 0.07 (< − 0.82–1.37)	2.51 (0.53–6.28)	0.39 (< − 0.43–1.91)	1.48 (− 0.046–4.55)	0.10 (< − 0.57–1.96)	1.38 (< − 0.46–5.95)
Changes in ERRs (%)	+ 61%	+ 12%	− 68%	− 48%	− 64%	− 46%
*Relative risk (95% CI)*^b^						
Low birth weight to normal birth weight	0.86 (0.44–1.66)		1.96 (1.18–3.25)		1.89 (0.84–4.22)	
Small head size to normal head size	0.61 (0.08–4.74)	0.76 (0.10–5.62)	2.16 (0.85–5.40)	3.02 (1.00–9.10)	2.33 (0.48–11.40)	3.81 (0.81–17.88)
Loss of father to father alive	0.74 (0.45–1.22)		1.51 (0.99–2.31)		1.14 (0.58–2.26)	
Loss of mother to mother alive	1.21 (0.57–2.57)		1.67 (0.89–3.16)		2.54 (1.03–6.24)	
*Attained age ≥ 20 years old*						
ERR/Gy (95% CI) Not adjusted for potential mediators^a^	− 0.18 (< − 0.77–0.94)	1.84 (0.18–4.98)	0.98 (− 0.09–2.98)	2.23 (0.078–7.13)	0.28 (< − 0.62–2.44)	2.75 (< − 0.45–10.52)
ERR/Gy (95% CI) Adjusted for potential mediators^b^	− 0.08 (< − 0.82–1.36)	2.10 (0.26–5.61)	0.52 (< − 0.44–2.34)	0.94 (− 0.56–4.33)	0.05 (< − 0.63–1.92)	1.59 (< − 0.56–7.20)
Changes in ERRs (%)	+ 58%	+ 14%	− 47%	− 58%	− 82%	− 42%
Relative risk (95% CI)^b^						
Low birth weight to normal birth weight	0.78 (0.39–1.56)		1.73 (0.98–3.05)		2.09 (0.92–4.75)	
Small head size to normal head size	0.62 (0.08–4.48)	0.83 (0.11–6.10)	1.43 (0.45–4.57)	4.13 (1.35–12.67)	2.25 (0.52–12.50)	5.56 (1.13–27.48)
Loss of father to father alive	0.75 (0.45–1.24)		1.45 (0.91–2.31)		1.07 (0.50–2.28)	
Loss of mother to mother alive	1.23 (0.58–2.60)		1.54 (0.75–3.18)		2.62 (0.94–7.33)	

^a^ERR/Gy was estimated based on the basic model, described as λ = λ_0_(c, s, c*nic, a, cs, t) * [1 + ERR(*d*)]

^b^ERR/Gy and relative risk at 1 Gy were estimated based on the full model, described as λ = λ_0_ (c, s, c*nic, a, cs, t, w, s*h, fs, ms) * [1 + ERR(*d*)]

λ: expected mortality rate, λ_0_: background rate, *c*: city, *s*: sex, *nic*: not in city at the time of atomic bombings, *a*: attained age, *cs*: cohort sources, *t*: trimester, *w*: birth weight, *h*: head size, *fs*: father’s survival status, and *ms*: mother’s survival status

#### Noncancer diseases

Among the all attained age groups, low birth weight was positively associated with background mortality for noncancer disease with a RR of 1.96 (95 CI: 1.18–3.25) (Table 5). The RR with small head size was 3.02 (95% CI: 1.00–9.10) in females and 2.16 (95% CI: 0.85–5.40) in males, and the RRs with loss of father and mother were 1.51 (95% CI: 0.99–2.31) and 1.67 (95% CI: 0.89–3.16), respectively. The unadjusted radiation-associated ERR/Gy of 1.22 (95% CI: 0.10–3.14) for males and 2.86 (95% CI: 0.56–7.64) for females were attenuated by 68% and by 48%, respectively, when adjusted for potential mediators, and these adjusted ERRs/Gy were no longer statistically significant (Table 5). The ERR/Gy for noncancer disease mortality did not differ by trimester at the time of bombing or attained age.

For adulthood noncancer disease mortality, small head size was positively associated with noncancer disease deaths in females (one case died from nervous system disease, two from circulatory disease, and one from respiratory disease). Those born with low birth weight and those who lost either parent or both parents had suggestive increase in RR. The unadjusted male ERR/Gy for noncancer disease mortality did not show strong evidence of radiation effects, while the unadjusted ERR/Gy for females was increased at 2.23 (95% CI: 0.078–7.13), but the adjusted ERR/Gy was attenuated by 58% and lost statistical significance (Table 5).

The unadjusted and adjusted ERRs/Gy for circulatory disease mortality in males and females were estimated but the confidence intervals were wide (Supplementary Table 2).

#### External causes

Among the all attained age groups, there was an increased background mortality rate in individuals who lost mother (RR = 2.54, 95% CI: 1.03–6.24). The ERR/Gy for external causes of death in males indicated no increase (0.28, 95% CI: < − 0.60–2.36), and neither did the adjusted ERR/Gy (0.10, 95% CI: < − 0.57–1.96) (Table 5). For females, an increased radiation-associated risk for external causes of death was suggested (ERR/Gy = 2.57, 95% CI: 0.20–9.19) but the adjusted ERR/Gy was attenuated by 46% (1.38, 95% CI: < − 0.45–5.95). The increased unadjusted ERRs/Gy for external causes of death among females seemed to be derived from suicide (the unadjusted ERR/Gy = 2.75, 95% CI: < − 0.89–15.63) because the unadjusted ERR/Gy for accidental deaths were not increased (ERR/Gy = − 0.08, < − 1.44–8.69), although both estimated confidence intervals were wide (Supplementary Table 3).

For adulthood mortality from external causes, small head size was positively associated with external causes of death among females (RR = 5.56, 95% CI: 1.13–27.48); individuals who were born with low birth weight (RR = 2.09, 95% CI: 0.92–4.75) and those who lost mothers (RR = 2.62, 95% CI: 0.94–7.33) had potentially raised mortality risks for external causes of death, although the lower bound of CIs was less than one. The unadjusted female radiation-associated ERR/Gy was raised but not statistically significant (2.75, 95% CI: < − 0.45–10.52), while the adjusted ERR/Gy decreased by 42% (1.59, 95% CI: < − 0.56–7.20). The ERR/Gy estimates for males were not increased (Table 5).

## Discussion

A significantly raised radiation-associated ERR/Gy of 2.24 (95% CI: 0.44–5.58) for solid cancer mortality in females exposed in utero was found for all attained ages. This estimate was somewhat smaller than that in the previous study (ERR/Sv = 3.3, 90% CI: 0.4–10.3) when considering only the in utero exposed group of individuals (i.e., excluding a group of Life Span Study members exposed to atomic bomb radiation in early childhood) followed until the age of 47 years in 1992 [1]; but the confidence interval was narrower in the present study. In males, no solid cancer deaths in exposed subjects were observed in the previous study, whereas 24 solid cancer deaths were observed in individuals exposed to radiation greater than 5 mGy in this study, although there was no increased radiation-associated risk for solid cancer in males. When cancer deaths of some sites and sex-specific sites were excluded from all solid cancer deaths, the ERR estimate in females was attenuated but still being increased. The number of cases was small even in the analysis for all solid cancer deaths, and exclusion of certain cases, which accounted for a large portion of all solid cancer, resulted in further reduction of efficiency for assessing the radiation effect (as indicated by unstable estimates with wider confidence intervals). We could not conclude which specific cancer affected the sex difference of radiation effects on solid cancer mortality. Among the Japanese general population, cancer incidence and mortality rates are usually higher in women than in men until the age of 50 years. Further follow-up is required to examine the lifetime radiation-associated risk of mortality among individuals exposed in utero.

Although we found an increased radiation-associated risk of adulthood solid cancer mortality in females, as in the previous study [1, 4], the findings are inconsistent with those in other studies. The dose–response of solid cancer incidence among Techa River residents who were exposed in utero (mean soft tissue dose: 4.4 mGy, maximum dose: 295 mGy) was close to zero [15]. Mayak workers’ children who were exposed in utero (mean estimated dose in utero: 54 mGy, maximum dose: 945 mGy) demonstrated no association between radiation exposure and mortality for either solid cancer (ERR/Gy = − 0.1, 95% CI: < − 0.1–4.1) or leukemia (ERR/Gy = − 0.8, 95% CI: < − 0.8–46.9) [16]. Both studies found no sex difference in radiation risk. The Urals Prenatally Exposed Cohort (UPEC) (i.e., in utero exposed members of Techa River and Mayak cohort combined and exposed chronically, mean estimated dose in utero: 14.1 mGy) showed no increased risks of incidence (RR/10 mGy of in utero dose = 0.99, 95% CI: 0.96–1.01) or mortality (RR/10 mGy of in utero dose = 0.98, 95% CI: 0.94–1.01) for adulthood solid cancer (the mean age among alive subjects at the end of follow up: 53 years) [5] and no increased risks of incidence or mortality for hematological malignancy (mean age among alive subjects at the end of follow up: 51 years) [6]. The authors of the UPEC study considered a limited number of cases because of the young age of the cohort and thus limited ability to estimate cancer risks.

In this study, an unadjusted association between noncancer disease mortality and radiation exposure was observed for all attained ages; however, the risks were attenuated in adulthood. This result was consistent with previous findings of high childhood mortality risks associated with closer distance to the hypocenter based on the socioeconomic survey conducted in 1964 [9], and non-elevated adult-onset noncancer disease risks associated with radiation dose among an in utero subcohort that was clinically followed [7]. In this study, the ERRs/Gy were also attenuated by 68% in males and 48% in females after adjustment for low birth weight (and other factors). This result suggests that low birth weight, among other factors (e.g., small head size), might be a crucial mediator between radiation exposure and childhood noncancer disease mortality.

Low birth weight was positively associated with noncancer disease mortality in adulthood. This evidence is consistent with the fetal origins of adult disease (FOAD) hypothesis [17–19]. To our knowledge, this study was the first to demonstrate such an association in the Japanese population, although another Japanese study reported that low birth weight was associated with an increase in blood pressure and serum cholesterol in adult life, which are established risk factors for CVD [20]. Small head size was also associated with an increased risk of noncancer disease mortality in females (but a weaker association in males). In an earlier clinical in utero cohort study by Wood, increased rates of noninfectious diseases and developmental abnormalities among children with intellectual disability and a small head, including two cases with Down syndrome [13], was suggested. Thus, low birth weight and small head size appear to be crucial risk factors for adult noncancer diseases.

Antenatal radiation exposure was associated with low birth weight in this study. Although experimental studies also demonstrated that mice exposed to high doses of radiation tended to give birth to low birth weight pups [21], there was no study to observe the association in human population except for a Chernobyl study [22]. The study found no relationship between low birth weight and in utero exposure to radioactive iodine-131 at fetal thyroid doses ranging between 0 and 2263 mGy (mean = 72 mGy), although the type and dose rate of radiation exposure following the Chernobyl accident were different from those for subjects of this study [22]. On the other hand, a systematic review suggested that mothers exposed to armed conflict had an increased risk of giving birth to babies with low birth weights because of insufficient medical care in the regions of the Gulf War, the al-Aqsa Intifada, Belgrade, Norway [23, 24], and Bosnia-Herzegovina [23]. A shortage of food and undernutrition likely contributed to the high prevalence of low birth weight [25], as observed in the Japanese population during the war period (regardless of radiation exposure) [26], the Dutch famine study [27], and the Leningrad siege study [28]. Mental stress during pregnancy has also been reported to increase the risk of low birth weight babies [24, 29]. Similarly in the situation after the atomic bombings, subjects’ mothers who were exposed within 2000 m of the hypocenters were thought to have sustained injury due to radiation and/or blast effects and had no access to medical support after the bombings, because 225 of 298 physicians died from the bombing in Hiroshima [30]. Therefore, the influence of physical injuries and social factors that were not measured in this study needs to be considered for the effects of atomic bombings.

We observed a higher risk of noncancer disease mortality among individuals who lost their father. This might suggest the compromised socioeconomic status and influence of poverty in families who lost their father. Communicable disease and inadequate sanitation facilities could also cause high childhood noncancer disease-associated mortality, as observed during armed conflict [23]. The radiation dose was not associated with the loss of mother; however, the loss of mother was a risk factor for external causes of death. The loss of father and mother should be considered when evaluating the radiation-associated risk of mortality for both noncancer disease and external causes of death in individuals exposed to the atomic bomb radiation in utero.

This study has several limitations. First, fetal radiation doses by gestational age at exposure were not available, thus maternal uterine doses were used as a surrogate. Recently, a collaborative study developed the J45 Pregnant Female Voxel Phantom Series scaling to 1945 Japanese body size to estimate fetal dose and uterine wall dose of a pregnant woman [31]. According to this report, dose to the uterine wall of the non-pregnant female generally underestimates fetal organ dose within the pregnant female. The magnitude of difference between these differences varies with both radiation type and irradiation geometry, with the smallest differences (5–7%) seen for isotropic photon fields and the largest differences (20–30%) seen for anterior–posterior neutron fields. As for the fetal whole body dose, the difference becomes larger at younger gestational age. Thus, there might be some bias of radiation risk estimate for individuals, particularly in the first trimester at the time of bombing. Second, we did not obtain lifestyle factors and socioeconomic status in adult life from our subjects. Third, head size information for individuals who died before the age of 9 years and for individuals derived only from the mortality program was not obtained. We assumed that all individuals with small head sizes were included in this study and that the remaining individuals had normal head size. However, 259 subjects of 715 exposed within 2000 m of the hypocenters were not included in the clinical program and belonged only to the mortality program. Thus, there might be a bias leading to an apparent reduction of mortality rates for individuals with small head size. Fourth, under the presence of unobserved confounders, total effect of radiation on the mortality risks could be biased due to adjustment for the possible mediators [32]. Although conditioning on a mediator is of concern in epidemiologic studies and indeed leads to bias in estimating the total effect of the exposure on the outcome, mediators and confounders cannot be distinguished in terms of statistical association [33]. We only showed some possible mediators and sensitivity of the ERR estimates due to the adjustment for them. Lastly, the subjects of this study have been followed since 1950 or later. Early mortality, especially for hematopoietic tumors with age peaks in the first years of life, could not be surveyed due to insufficient information. Several efforts by the mortality program were made retrospectively to assess mortality from birth to the age of 19 years. For example, information of cause of deaths for one third of the deceased subjects (primarily during first few years after bombings) could not be obtained because the information of death certificate was not always kept for long time in the public health centers. The proportion of cause-specific deaths to total deaths with cause known and the cause-specific death rates during 18 years after birth among in utero subjects of the mortality program were reported by Kato et al. [9]. In spite of these limitations, this cohort will be able to provide useful information on the radiation-associated risk of mortality for those exposed in utero due to lifelong follow-up [15, 16, 22].

## Conclusion

This study provided three essential findings. First, radiation-associated risk of solid cancer mortality was increased among female in utero survivors, regardless of adjustment for potential mediators, birth weight, head size, and parental survival status; but this was not found for males. Second, the radiation-associated risk for noncancer disease mortality was increased in both sexes, but adjustment for potential mediators explained the radiation-associated excess risk. Third, exposure to atomic bomb radiation seemed to affect female mortality from external causes through the loss of mother and female mortality in adulthood due to small head size. Further follow-up is required to examine the lifetime radiation-associated risk of mortality among individuals exposed in utero. Further efforts are also required to determine important mediators by using advanced modeling or better surrogate measure to assess mortality among individuals exposed in utero to atomic bombing.

## Electronic supplementary material

Below is the link to the electronic supplementary material.

Supplementary material 1 (DOCX 28 kb)

## Data Availability

Code is not provided.
