# Supplementary material for: Mortality among individuals exposed to atomic bomb radiation in utero: 1950–2012
Source: Eur J Epidemiol. 2021 Jan 25;36(4):415–28. doi: 10.1007/s10654-020-00713-5 (PMC8076150; doi:10.1007/s10654-020-00713-5)
Supplement: Supplementary file 1 — Supplementary material 1 (DOCX 28 kb) [file 10654_2020_713_MOESM1_ESM.docx]

Supplementary Table 1. The unadjusted ERR/Gy for adulthood solid cancer mortality by selected cancer site and those excluded selected cancer sites.

|  | ERR/Gy (95% CI) | |  | ERR/Gy (95% CI) | |
| --- | --- | --- | --- | --- | --- |
| Selected cancer site | Males | Females | Excluded cancer site | Males | Females |
| All solid cancer deaths | -0.18 | 1.84 | All solid cancer deaths | -0.18 | 1.84 |
|  | (<-0.77 to 0.94) | (0.18 to 4.98) |  | (<-0.77 to 0.94) | (0.18 to 4.98) |
| Stomach cancer deaths | 0.06 | 7.10 | excluding stomach cancer deaths | Fixed to be 0 ^a)^ | 1.41 |
|  | (<-0.85 to 2.67) | (<-3.79 to 50.63) |  |  | (-0.06 to 4.46) |
| Lung cancer deaths | No convergence ^b)^ | No convergence ^b)^ | excluding lung cancer deaths | -0.04 | 2.18 |
|  |  |  |  | (<-0.82; 1.42) | (0.29; 5.82) |
| Liver cancer deaths | 0.21 | 6.28 | excluding liver cancer deaths | -0.20 | 1.41 |
|  | (<-2.44 to 7.50) | (<-3.38 to 61.84) |  | (<-0.78; 1.02) | (-0.07 to 4.37) |
| Breast cancer deaths | -0.39 | 2.91 | excluding breast cancer deaths | -0.16 | 1.48 |
|  | (＜-0.80 to >2573 ) | (<1.22 to >6.18) |  | (<-0.78 to 1.01) | (-0.07 to 4.65) |
| Sex-specific cancers deaths ^c)^ | Fixed to be 0. ^a)^ | 2.58 | excluding sex-specific cancers deaths ^c)^ | -0.15 | 1.03 |
|  |  | (<-0.61 to 10.42) |  | (<-0.78 to 1.06) | (<-0.74 to 4.46) |
| ERR: Excess relative risk. CI: Confidence intervals. | |  |  |  |  |
| a) The ERR model did not converge. The male ERR/Gy was fixed to be zero. | | |  |  |  |
| b) The ERR model did not converge. The relative risks/Gy for males and females were estimated to be 0.11 (95% CI: 0.00 to 15.23) and 0.44 (95% CI: 0.00 to 83.44), respectively. | | | | | |
| c) Sex-specific cancers included cancers for breast, vulva, vagina, uterus, ovary, and prostate. | | | | | |

Supplementary Table 2. Radiation ERRs/Gy and relative risks of potential mediators on circulatory disease mortality among individuals exposed to atomic bomb radiation *in utero*

| All attained age | | Circulatory disease | |
| --- | --- | --- | --- |
|  |  | Male | Female |
| ERR/Gy (95% CI) | | 1.18 | 2.72 |
| Not adjusted for potential mediators ^a)^ | | (<-0.74 to 5.37) | (<-0.89 to 13.12) |
| ERR/Gy (95% CI) | | -0.10 | 1.38 |
| Adjusted for potential mediators ^b)^ | | (<-1.53 to 3.24) | (<-1.72 to 9.63) |
| Changes in ERRs (%) | | -108% | -49% |
| Relative risk (95% CI) ^b)^ | |  |  |
| Low birth weight | | 1.45 | |
| to normal birth weight | | (0.62 to 3.42) | |
| Small head size | | 2.91 | 3.44 |
| to normal head size | | (0.73 to 11.66) | (0.70 to 16.88) |
| Loss of father | | 1.44 | |
| to father alive | | (0.71 to 2.90) | |
| Loss of mother | | 0.29 | |
| to mother alive | | (0.04 to 2.37) | |
| Attained age >=20 years old | |  |  |
| ERR/Gy (95% CI) | | 0.75 | 2.66 |
| Not adjusted for potential mediators ^a)^ | | (<-1.23 to 4.53) | (<-0.89 to 12.89) |
| ERR/Gy (95% CI) | | 0.06 | 1.59 |
| Adjusted for potential mediators ^b)^ | | (<-1.53 to 3.62) | (-1.79 to 10.46) |
| Changes in ERRs (%) | | -92% | -40% |
| Relative risk (95% CI) ^b)^ | |  |  |
| Low birth weight | | 1.27 | |
| to normal birth weight | | (0.51 to 3.15)) | |
| Small head size | | 1.83 | 3.58 |
| to normal head size | | (0.35 to 9.53) | (0.73 to 17.48) |
| Loss of father | | 1.30 | |
| to father alive | | (0.63 to 2.69) | |
| Loss of mother | | 0.29 | |
| to mother alive | | (0.04 to 2.33) | |
| ERR: Excess relative risk. CI: Confidence intervals. | | |  |

Supplementary Table 3. Radiation ERRs/Gy and relative risks of potential mediators on suicide and accidental mortality among individuals exposed to atomic bomb radiation *in utero*

| All attained age | | Suicide | | Accidental deaths | |
| --- | --- | --- | --- | --- | --- |
|  |  | Male | Female | Male | Female |
| ERR/Gy (95% CI) | | 0.21 | 2.75 | 0.57 | -0.08 |
| Not adjusted for potential mediators ^a)^ | | (<-0.69 to 2.72) | (<-0.89 to 15.63) | (<-1.97 to 7.74) | (<-1.44 to 8.69) |
| ERR/Gy (95% CI) | | 0.15 | 1.47 | 1.04 | Fixed to be 0. ^a)^ |
| Adjusted for potential mediators ^b)^ | | (<-0.68 to 2.65) | (<-0.79 to 10.59) | (<-2.21 to 10.09) |  |
| Changes in ERRs (%) | | -30% | -47% | 82% | - |
| Relative risk (95% CI) ^b)^ | |  |  |  |  |
| Low birth weight | | 1.87 | | 0.60 | |
| to normal birth weight | | (0.59 to 5.88) | | (0.08 to 4.55) | |
| Small head size | | 2.49 | 4.77 | 0.00 | 5.24 |
| to normal head size | | (0.28 to 22.36) | (0.52 to 44.06) | - | (0.59 to 46.16) |
| Loss of father | | 0.97 | | 1.24 | |
| to father alive | | (0.36 to 2.62) | | (0.43 to 3.58) | |
| Loss of mother | | 3.24 | | 1.69 | |
| to mother alive | | (0.97 to 10.80) | | (0.37 to 7.76) | |
| Attained age >=20 years old | |  |  |  |  |
| ERR/Gy (95% CI) | | 0.22 | 2.60 | Not converged ^b)^ | |
| Not adjusted for potential mediators ^a)^ | | (<-0.69 to 2.77) | (<-1.02 to 14.84) |  |  |
| ERR/Gy (95% CI) | | 0.14 | 1.37 | Not converged ^b)^ | |
| Adjusted for potential mediators ^b)^ | | (<-0.67 to 2.60) | (<-0.87 to 9.90) |  |  |
| Changes in ERRs (%) | | -38% | -47% |  |  |
| Relative risk (95% CI) ^b)^ | |  |  |  |  |
| Low birth weight | | 1.97 | |  | |
| to normal birth weight | | (0.62 to 6.26) | |  | |
| Small head size | | 2.39 | 5.19 |  |  |
| to normal head size | | (0.26 to 21.65) | (0.55 to 49.10) |  |  |
| Loss of father | | 1.09 | |  | |
| to father alive | | (0.40 to 2.96) | |  | |
| Loss of mother | | 2.25 | |  | |
| to mother alive | | (0.59 to 8.60) | |  | |
| ERR: Excess relative risk. CI: Confidence intervals. | | |  |  |  |
| a) The ERR model did not converge. The female ERR/Gy was fixed to be zero. | | | |  |  |
| b) The ERR model did not converge. | |  |  |  |  |
